# Trajectories of forced vital capacity in patients with systemic sclerosis-associated interstitial lung disease

**DOI:** 10.1186/s13075-025-03524-9

**Published:** 2025-03-21

**Authors:** Oliver Distler, Madelon C Vonk, Arata Azuma, Maureen D. Mayes, Dinesh Khanna, Kristin B. Highland, Gerrit Toenges, Margarida Alves, Yannick Allanore

**Affiliations:** 1 Department of Rheumatology, University Hospital Zurich, University of Zurich, Zurich, Switzerland; 2 Department of Rheumatology, Radboud University Medical Center, Nijmegen, The Netherlands; 3 Pulmonary Medicine and Clinical Research Centre, Mihara General Hospital, Saitama, Japan; 4 Nippon Medical School, Tokyo, Japan; 5 Division of Rheumatology, University of Texas McGovern Medical School, Houston, Texas USA; 6 Department of Medicine, University of Michigan Scleroderma Program, University of Michigan, Ann Arbor, Michigan USA; 7 Cleveland Clinic, Cleveland, Ohio USA; 8 Boehringer Ingelheim Pharma GmbH & Co. KG, Ingelheim am Rhein, Germany; 9 Boehringer Ingelheim International GmbH, Ingelheim, Germany; 10 Department of Rheumatology A, Descartes University, APHP, Cochin Hospital, Paris, France

**Keywords:** Disease progression, Pulmonary fibrosis, Pulmonary function tests, Systemic scleroderma

## Abstract

We used data from the SENSCIS and SENSCIS-ON trials to assess decline in forced vital capacity (FVC) in patients with systemic sclerosis-associated interstitial lung disease (SSc-ILD) who received long-term treatment with nintedanib and the effect of switching patients from placebo to nintedanib. In the SENSCIS trial, patients were randomised to receive nintedanib or placebo until the last patient reached week 52 but for ≤ 100 weeks. In SENSCIS-ON, the extension to SENSCIS, all patients received open-label nintedanib. Per protocol, the off-treatment period between these trials was ≤ 12 weeks. We assessed the trajectory of FVC in patients who received nintedanib in SENSCIS and continued nintedanib in SENSCIS-ON (*n* = 197) and in patients who received placebo in SENSCIS and initiated nintedanib in SENSCIS-ON (*n* = 231). The last on-treatment measurement in SENSCIS and the baseline measurement of SENSCIS-ON were considered anchor measurements. In patients who received nintedanib in SENSCIS, the mean decline in FVC in the 52 weeks prior to the last on-treatment measurement in SENSCIS was − 41.5 mL and the mean decline in FVC from baseline to week 52 of SENSCIS-ON was − 58.3 mL. In patients who received placebo in SENSCIS, the mean decline in FVC in the 52 weeks prior to the last on-treatment measurement in SENSCIS was − 96.8 mL and the mean decline in FVC from baseline to week 52 of SENSCIS-ON (when patients received nintedanib) was − 42.8 mL. These findings illustrate the progressive nature of SSc-ILD and support the efficacy of nintedanib in slowing decline in lung function over the long term.

## Introduction

Interstitial lung disease (ILD) is a common manifestation of systemic sclerosis (SSc) [1, 2]. The progression of SSc-associated ILD (SSc-ILD) may be assessed through serial measurement of forced vital capacity (FVC) [3, 4]. A decline in FVC in patients with SSc-ILD is associated with an increased risk of hospitalisation and mortality [1, 5–8].

The SENSCIS trial enrolled patients with SSc and fibrotic ILD without a requirement for recent progression [9]. Patients were randomised to receive nintedanib or placebo. Patients who completed the SENSCIS trial were eligible to enter an extension trial known as SENSCIS-ON, in which all received open-label nintedanib [10]. The primary data on adverse events and decline in FVC in patients treated with nintedanib in SENSCIS and SENSCIS-ON have been published [9, 10]. It would be expected that patients who switched from placebo to open-label nintedanib would then have a similar rate of decline in FVC as observed in patients initially randomised to receive nintedanib. In this analysis, we used an anchor-based method to assess the trajectories of FVC decline in the treatment periods of SENSCIS and SENSCIS-ON to investigate FVC decline in patients with SSc-ILD treated with nintedanib over the longer term and the effect of switching patients with SSc-ILD from placebo to nintedanib.

## Methods

Patients in the SENSCIS trial had SSc with first non-Raynaud symptom in the prior ≤ 7 years, an extent of fibrotic ILD on high-resolution computed tomography ≥ 10%, FVC ≥ 40% predicted and diffusing capacity of the lung for carbon monoxide (DLco) 30–89% predicted. Patients were randomised to receive nintedanib 150 mg twice daily (bid) or placebo, stratified by anti-topoisomerase I antibody status. Patients remained on blinded treatment until the last patient had reached week 52 but for ≤ 100 weeks. Patients who completed the SENSCIS trial on trial medication and attended a follow-up visit 28 days later were eligible to enter SENSCIS-ON. Per protocol, the off-treatment period between SENSCIS and SENSCIS-ON was ≤ 12 weeks.

In both SENSCIS and SENSCIS-ON, FVC was measured using sponsor-supplied spirometers and in accordance with American Thoracic Society/European Respiratory Society guidelines [11]. FVC was measured at baseline and at weeks 4, 12, 24, 36, 52, 68, 84 and 100 of both the SENSCIS and SENSCIS-ON trials. FVC was also measured at weeks 2 and 6 of the SENSCIS trial.

**Fig. 1:**
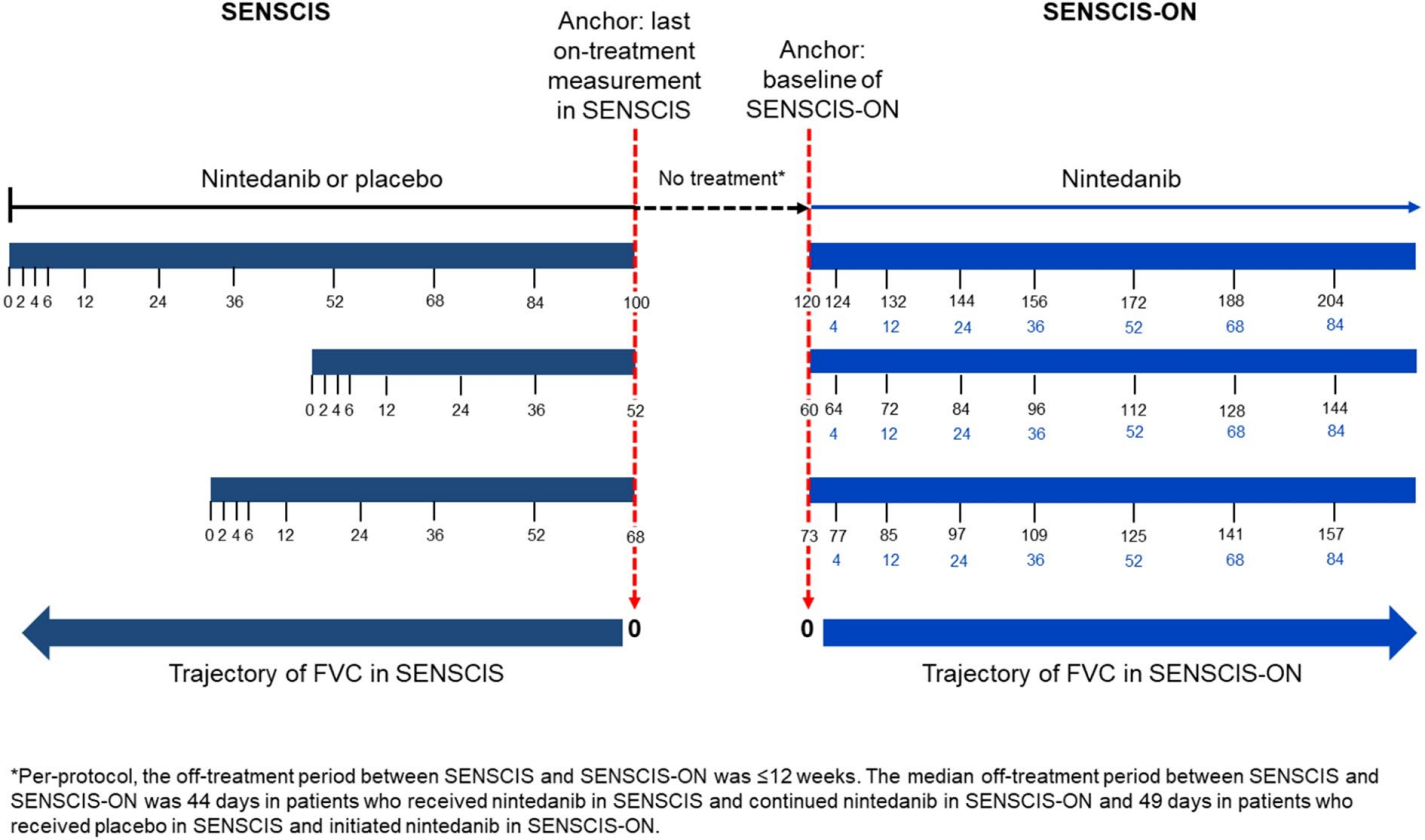
Measuring the trajectories of FVC in SENSCIS and SENSCIS-ON

Trajectories of FVC were assessed in two groups: patients who received nintedanib in SENSCIS and continued nintedanib in SENSCIS-ON and patients who received placebo in SENSCIS and initiated nintedanib in SENSCIS-ON. As the off-treatment period between SENSCIS and SENSCIS-ON varied between patients, the change in FVC was calculated based on two anchors. The last on-treatment measurement in SENSCIS was used as the anchor to calculate the change in FVC in a backward direction (i.e. during the on-treatment period of SENSCIS) and the baseline measurement in SENSCIS-ON was used as the anchor to calculate the change in FVC in a forward direction *(i.e.*. in SENSCIS-ON) (Fig. 1). This approach allows a direct comparison between the on-treatment FVC declines in SENSCIS and SENSCIS-ON. Analyses were descriptive and based on observed values.

## Results

In total, 197 patients received nintedanib in SENSCIS and continued nintedanib in SENSCIS-ON; 231 patients received placebo in SENSCIS and initiated nintedanib in SENSCIS-ON. In patients who received nintedanib in SENSCIS and continued nintedanib in SENSCIS-ON, mean (SE) FVC was 72.3 (1.2) % predicted and 2481 (52.8) mL at the start of SENSCIS, 70.9 (1.3) % predicted and 2430 (55.1) mL at the last on-treatment measurement in SENSCIS, and 70.4 (1.3) % predicted and 2379 (60.3) mL at the start of SENSCIS-ON. In patients who received placebo in SENSCIS and initiated nintedanib in SENSCIS-ON, mean (SE) FVC was 73.7 (1.1) % predicted and 2593 (54.8) mL at the start of SENSCIS, 70.5 (1.1) % predicted and 2472 (52.4) mL at the last on-treatment measurement in SENSCIS and 70.2 (1.2) % predicted and 2441 (55.3) mL at the start of SENSCIS-ON. The median off-treatment period between SENSCIS and SENSCIS-ON was 44 days in patients who received nintedanib in SENSCIS and continued nintedanib in SENSCIS-ON and 49 days in patients who received placebo in SENSCIS and initiated nintedanib in SENSCIS-ON.

**Fig. 2:**
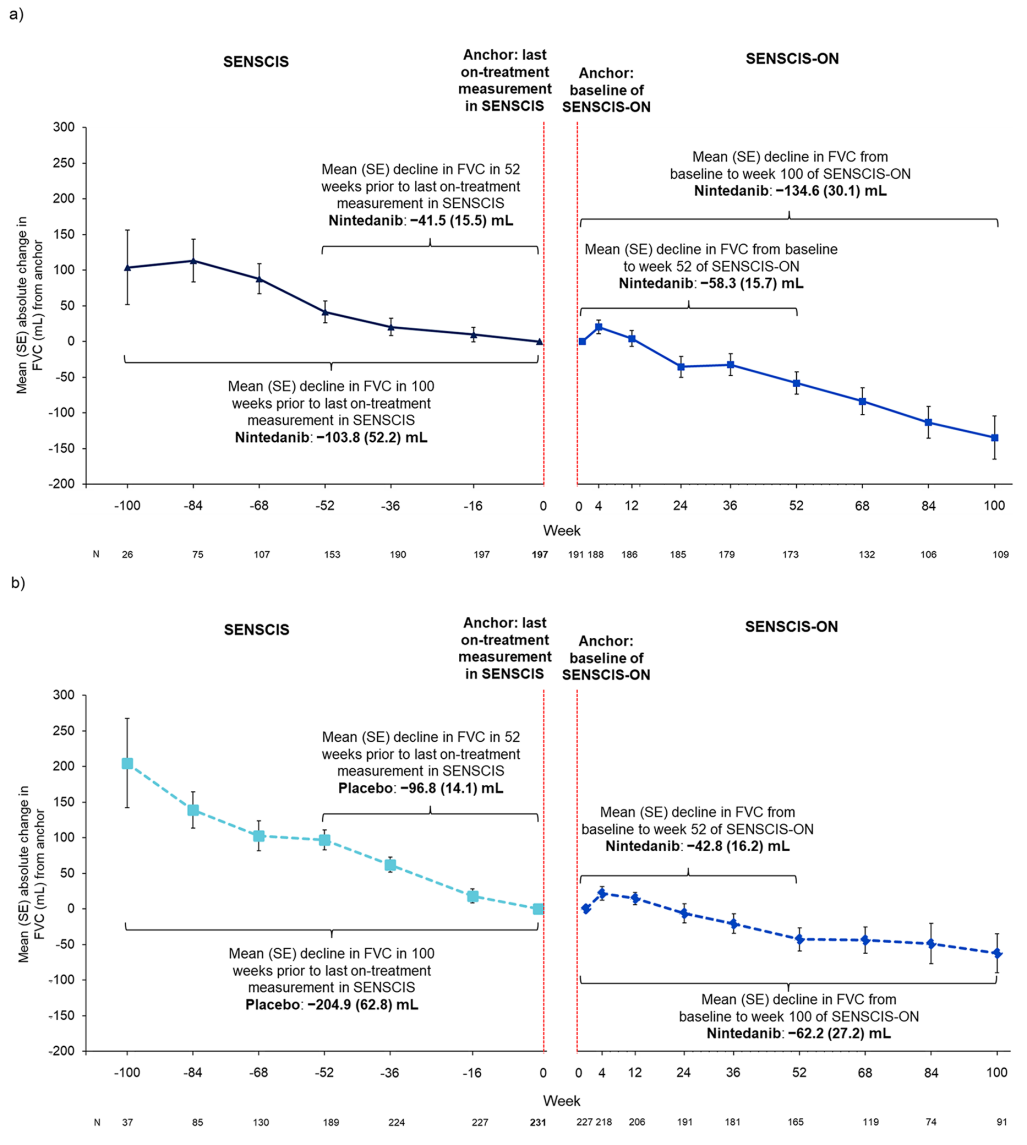
Trajectories of FVC in SENSCIS and SENSCIS-ON in (**a**) patients who received nintedanib in SENSCIS and (**b**) patients who received placebo in SENSCIS

In patients who received nintedanib in SENSCIS, the mean (SE) decline in FVC in the 52 weeks prior to the last on-treatment measurement in SENSCIS was − 41.5 (15.5) mL (*n* = 153) and the mean (SE) decline in FVC from baseline to week 52 of SENSCIS-ON was − 58.3 (15.7) mL (*n* = 173) (Fig. 2a). In this group, the mean (SE) decline in FVC in the 100 weeks prior to the last on-treatment measurement in SENSCIS was − 103.8 (52.2) mL (*n* = 26) and the mean (SE) decline in FVC from baseline to week 100 of SENSCIS-ON was − 134.6 (30.1) mL (*n* = 109) (Fig. 2a).

In patients who received placebo in SENSCIS, the mean (SE) decline in FVC in the 52 weeks prior to the last on-treatment measurement in SENSCIS was − 96.8 (14.1) mL (*n* = 189) and the mean (SE) decline in FVC from baseline to week 52 of SENSCIS-ON (when patients were receiving nintedanib) was − 42.8 (16.2) mL (*n* = 165) (Fig. 2b). In this group, the mean (SE) decline in FVC in the 100 weeks prior to the last on-treatment measurement in SENSCIS was − 204.9 (62.8) mL (*n* = 37) and the mean (SE) decline in FVC from baseline to week 100 of SENSCIS-ON of − 62.2 (27.2) mL (*n* = 91) (Fig. 2b).

## Discussion

We assessed the trajectories of FVC in patients with SSc-ILD in the on-treatment periods of the SENSCIS and SENSCIS-ON trials. In the SENSCIS trial, consistent with the findings of previous analyses [9, 12–14], progression of SSc-ILD was observed in both treatment groups, but the decline in FVC was smaller in the nintedanib group than in the placebo group. Among patients who had received placebo in the SENSCIS trial, the decline in FVC over 52 weeks following initiation of open-label nintedanib in SENSCIS-ON was reduced to a rate of decline that was similar to that observed in the patients who received nintedanib in the SENSCIS trial, supporting the efficacy of nintedanib. In patients who received nintedanib in the SENSCIS trial, the declines in FVC over 52 weeks in SENSCIS and SENSCIS-ON were similar, suggesting a continued effect of nintedanib. An earlier analysis of pooled data from SENSCIS and SENSCIS-ON, in which time zero was regarded as the start of the SENSCIS trial, also suggested a sustained effect of nintedanib on slowing decline in FVC [10]. The current analysis, which used an anchor-based approach to account for the variability in the duration of the SENSCIS trial and the off-treatment period between the trials, may provide a more comprehensive picture of the effect of nintedanib, particularly among patients who received placebo in SENSCIS and initiated nintedanib in SENSCIS-ON.

Several studies have demonstrated an association between decline in FVC and mortality in patients with SSc-ILD [1, 5–8]. The findings of Delphi consensus panels, as well as clinical practice guidelines issued by the American Thoracic Society (ATS) and American College of Rheumatology/American College of Chest Physicians, highlight the importance of monitoring patients with SSc-ILD for progression and of taking action to slow the progression of the disease [3, 4, 15, 16]. The ATS and ACR guidelines provide conditional recommendations for the use of nintedanib in patients with SSc-ILD [15, 17]. An international clinical practice guideline issued by respiratory societies in 2022 provided a conditional recommendation for the use of nintedanib in patients with progressive pulmonary fibrosis (other than idiopathic pulmonary fibrosis) who have failed standard management [18]; however, to wait for patients with SSc-ILD to meet these criteria for progression would miss the opportunity to treat patients with early and slowly progressing disease, for whom the benefits of treatment might be the greatest [19].

Limitations of these analyses include the gradual loss of patients over the course of the trials. There may have been selection bias among the patients who participated in, and continued in, SENSCIS-ON, in that these patients may have had better lung function or slower progression or been better able to tolerate nintedanib. Comparisons between patients who continued nintedanib and initiated nintedanib in SENSCIS-ON should be approached with caution given that patients were not randomized on entry into SENSCIS-ON.

In conclusion, the trajectories of decline in FVC in the SENSCIS and SENSCIS-ON trials illustrate the progressive nature of SSc-ILD in the population studied and support the efficacy of nintedanib on slowing decline in lung function in these patients over the longer term.

## Data Availability

To ensure independent interpretation of clinical study results and enable authors to fulfil their role and obligations under the ICMJE criteria, Boehringer Ingelheim grants all authors access to relevant clinical study data. In adherence with the Boehringer Ingelheim Policy on Transparency and Publication of Clinical Study Data, scientific and medical researchers can request access to clinical study data, typically one year after the approval has been granted by major regulatory authorities or after termination of the development programme. Researchers should use https://vivli.org/ to request access to study data and visit https://www.mystudywindow.com/msw/datasharing for further information.
